# Editorial: Serum uric acid, vascular aging, and endocrine comorbidities

**DOI:** 10.3389/fendo.2023.1292015

**Published:** 2023-09-26

**Authors:** Yijun Hu, Arrigo F.G. Cicero

**Affiliations:** ^1^ Guangdong Eye Institute, Department of Ophthalmology, Guangdong Provincial People’s Hospital (Guangdong Academy of Medical Sciences), Southern Medical University, Guangzhou, Guangdong, China; ^2^ Hypertension and Cardiovascular Risk Factors Research Centre, Medical and Surgical Sciences Department, Alma Mater Studiorum University of Bologna, Bologna, Italy; ^3^ Cardiovascular Medicine Unit, Heart-Chest-Vascular Department, IRCCS Azienda Ospedaliero-Universitaria di Bologna (AOUBO), Bologna, Italy

**Keywords:** uric acid, aging, vascular health, comorbidities, pathophysiology

It is largely known that uric acid is the final product of purine metabolism, and that its increased serum levels have been directly involved in the pathogenesis and natural history of a number of endocrino-metabolic and cardiovascular diseases (1).

In particular, serum uric acid (SUA) has been associated with the risk of developing hypertension and subclinical vascular damage (2), as well as an increased risk of myocardial infarction, stroke, heart failure, and different arrhythmias (in particular atrial fibrillation) by affecting thickness, function, and other features of the vascular tissue. As regards metabolic diseases, SUA is not only associated with a risk of gout, but also metabolic syndrome, non-alcoholic fatty liver disease, obesity, and type 2 diabetes. The risk of type 2 diabetes macrovascular and microvascular complications are also associated with increased SUA levels.

Meanwhile, suboptimal SUA levels have been associated with an increased risk for cardiovascular disease mortality, in particular in type 2 diabetes patients. This suggests a use for SUA levels as markers of vascular injury and vascular related diseases, which in turn can be associated with endocrine comorbidities.

But serum uric acid is not only related to insulin-resistance and type 2 diabetes. Since the 70s it was known the relationship between thyroid and parathyroid disorders and hyperuricemia (3). However, the association between uric acid levels, thyroid/parathyroid function and vascular damage has not yet deeply investigated.

In this context, Frontiers in Endocrinology hosted a Research Topic on serum uric acid, vascular aging and endocrine comorbidities. Li et al. observed in a large cohort of Chinese patients, that SUA levels are strongly related with metabolic syndrome and its component, but not with early sign of carotid aging in patients affected by type 2 diabetes. This data is partly in contrast with what known in general population, but patients with type 2 diabetes could have an increased vascular aging independently from other factors. On the other side, always in this Research Topic, Gao et al. showed in a different patients cohort with different degree of glucose-metabolism impairment, that carotid aging is more relevant in hyperuricemic subjects than in the normouricemic ones, more relevant in subjects affected by impaired fasting glucose and (even more) in diabetic ones. These evidences suggest a strong, but complex relationship between serum uric acid, glucose impairment and carotid aging. Less investigated is the nexus between serum uric acid and microvascular damage. In this Research Topic, Yang et al. interestingly suggested that serum uric acid is associated with a lower retinal capillary plexus measured by optical coherence tomography angiography in a large cohort of Chinese subjects. A similar trend has been recently observed as regards the relationship between serum uric acid and hypertensive white matter hyperintensity in dementia and stroke free patients (4). On the other side, serum uric acid association with microvascular damage could be tissue-specific, since it has not been observed in skin (5).

Among others, the large URRAH study identified SUA cut-off largely lower than the ones usually considered a risk factor for the development of gout (6, 7).

Beyond the rapidly increasing evidence relating SUA levels with all these risk factors, diseases, and mortality, more data are yet needed to clearly define the SUA cut-off associated with different outcomes in different populations and patients’ groups, where SUA is pathogenetic or an epiphenomenon, and the role of SUA-lowering therapies to prevent SUA related damages.

In this Research Topic, Hu et al. discovered a nonlinear relationship between SUA and phase angle (PhA) among type 2 diabetes mellitus patients. The PhA was increased with elevated SUA within a certain range, and the effect was insignificant beyond a specific threshold. These results suggest that SUA may function as a dual indicator of health and disease. Below a certain threshold, SUA can serve as a nutritional marker and antioxidant. However, above that threshold, SUA is harmful as an oxidant that accelerates tissue damage. In a cohort of Chinese patients with chronic heart failure (CHF), Wang et al. found that hyperuricemia and chronic kidney disease (CKD) significantly increased the risk of in-hospital mortality and long-term mortality. The evidence presented in this study showed the impact of both hyperuricemia and CKD on heart failure outcomes, emphasizing the importance of managing SUA in CHF patients. Additionally, a large cohort study conducted in the Korean population by Kang et al. found that patients with gout had a slightly higher incidence of stroke, ischemic heart disease, or heart failure compared to control subjects. This study demonstrated that gout was an independent risk factor for cardiovascular disease. On the other hand, a cross-sectional analysis conducted by Shen et al. indicated that low levels of urinary iodine concentration were significantly associated with a decreased prevalence of metabolic disorders and associated diseases in American participants. This association could potentially introduce novel dietary interventions to treat patients with metabolic disorders.

Many other interesting evidences are included in this Research Topic, suggesting the interesting interrelationship between endocrinopathies, serum uric acid, and vascular health.

## Author contributions

AC: Conceptualization, Writing – original draft, Writing – review & editing. YH: Writing – original draft, Writing – review & editing.
